# Porous metal materials for applications in orthopedic field: A review on mechanisms in bone healing

**DOI:** 10.1016/j.jot.2024.08.003

**Published:** 2024-10-11

**Authors:** Yutong Ma, Yi Wang, Shuang Tong, Yuehan Wang, Zhuoya Wang, Rongze Sui, Ke Yang, Frank Witte, Shude Yang

**Affiliations:** a Department of Plastic Surgery, The First Hospital of China Medical University, Shenyang, 110001, China; b Department of Breast Surgery, The First Hospital of China Medical University, Shenyang, 110001, China; c The First Clinical College of China Medical University, Shenyang, 110001, China; d Institute of Metal Research, Chinese Academy of Sciences, Shenyang, 110016, China; e Department of Prosthodontics, Geriatric Dentistry and Craniomandibular Disorders, Charité Medical University, Assmannshauser Strasse 4–6, 14197, Berlin, Germany

**Keywords:** Angiogenesis, Bone regeneration, Immunomodulation, Osteogenesis, Porous metal materials

## Abstract

**Background:**

Porous metal materials have been widely studied for applications in orthopedic field, owing to their excellent features and properties in bone healing. Porous metal materials with different compositions, manufacturing methods, and porosities have been developed. Whereas, the systematic mechanisms on how porous metal materials promote bone healing still remain unclear.

**Methods:**

This review is concerned on the porous metal materials from three aspects with accounts of specific mechanisms, inflammatory regulation, angiogenesis and osteogenesis. We place great emphasis on different cells regulated by porous metal materials, including mesenchymal stem cells (MSCs), macrophages, endothelial cells (ECs), etc.

**Result:**

The design of porous metal materials is diversified, with its varying pore sizes, porosity material types, modification methods and coatings help researchers create the most experimentally suitable and clinically effective scaffolds. Related signal pathways presented from different functions showed that porous metal materials could change the behavior of cells and the amount of cytokines, achieving good influence on osteogenesis.

**Conclusion:**

This article summarizes the current progress achieved in the mechanism of porous metal materials promoting bone healing. By modulating the cellular behavior and physiological status of a spectrum of cellular constituents, such as macrophages, osteoblasts, and osteoclasts, porous metal materials are capable of activating different pathways and releasing regulatory factors, thus exerting pivotal influence on improving the bone healing effect.

**The translational potential of this article:**

Porous metal materials play a vital role in the treatment of bone defects. Unfortunately, although an increasing number of studies have been concentrated on the effect of porous metal materials on osteogenesis-related cells, the comprehensive regulation of porous metal materials on the host cell functions during bone regeneration and the related intrinsic mechanisms remain unclear. This review summarizes different design methods for porous metal materials to fabricate the most suitable scaffolds for bone remodeling, and systematically reviews the corresponding mechanisms on inflammation, angiogenesis and osteogenesis of porous metal materials. This review can provide more theoretical framework and innovative optimization for the application of porous metal materials in orthopedics, dentistry, and other areas, thereby advancing their clinical utility and efficacy.

## Graphical abstract

**Figure:**
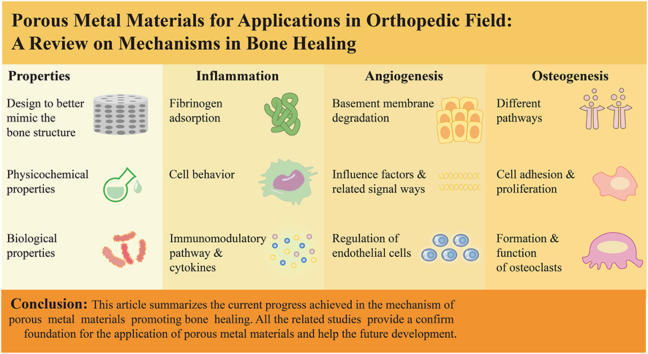

## 1 Introduction

It has been known that bone regeneration is an essential and fixed part of the bone remodeling. Not all diseases can be cured by only patients’ own bone regeneration process, especially in some complicated clinical conditions where bone regeneration could not bring the bone back to the normal state on itself. It has been reported that large bone defects created by osteomyelitis trauma [1], comminuted fracture [2]，different kinds of tumor metastasis [3] and infection [4] hinder the healing beginning. Or the regenerative process is compromised, including avascular necrosis [5], atrophic non-unions and osteoporosis [6]. Therefore, a large number of materials has been developed to solve these problems. Stainless steels, titanium and its alloys, and cobalt-based alloys are traditional orthopedic implants materials widely used, exhibiting superior biocompatibility, chemical inertness, and mechanical properties [7]. Nevertheless, they also have the non-ignorable disadvantages, such as infecting and loosening of the implants. Their modulus is higher than those of human bones including cancellous bone and cortical bone. The mismatch may lead to a stress shielding effect, which can cause serious complications such as periprosthetic fractures during or after the repair surgery and aseptic loosening as well [8]. The reason is that the traditional orthopedic implants without apertures have poor adsorption capacity and angiogenic ability. They inhibit cellular invasion, cell proliferation and extracellular matrix formation, which may ultimately lead to the implantation failure. Similarly, their badly-matched elastic modulus with bone tissue may also cause the same result. Therefore, a second surgery is often needed on account of the above problems.

In contrast, porous materials can mimic the architecture of the native bone and possess acceptable levels of bioactivity, biocompatibility, even biodegradability, and structural integrity. What's more, the porous structure also provides space for inward growth of the bone to achieve the osseointegration. Porous materials with interconnected and suitable pores can also help to increase cell adsorption [9], and promote the distribution of nutrients by newly formed blood vessels [10] as well as osteogenic ability [11]. Meanwhile, they could decrease the undesired stress shielding effect [12]. In this case, a second surgery becomes redundant.

The categories of porous materials can be roughly divided into two kinds, metals and nonmetals. The characters of them differ a lot, which cannot be stated synthetically. Therefore, only the porous metal materials are emphasized in this review.

In order to better mimic the bone marrow structure and create an immune microenvironment suitable for cell survival, the design of porous metal materials is apparently important, including the pore size，bioactive coatings, material categories [13], manufacturing methods [14], anisotropy [15], topology [16], and so on. The exact description is in the Chapter Two. A variety of studies on porous metal materials such as porous titanium and porous tantalum were conducted. Additionally, the spatial structure of the porous metal material also has an effect on cell growth, while the irregular porous structure is usually better than the regular porous structure.

More theoretical basis is given through the exploration of the mutual effects and the potential mechanisms between porous metal materials and the host after implantation, aiming at the prioritization of porous metal materials. To move forward a single step, promoting the application of porous metal materials in orthopedic field is promising. Unfortunately, although the effect of porous metal materials on bone regeneration-related cells and signal ways is drawing more and more attention, the exact mechanism of how bone formation and angiogenesis are regulated still keeps unknown.

**Figure 1:**
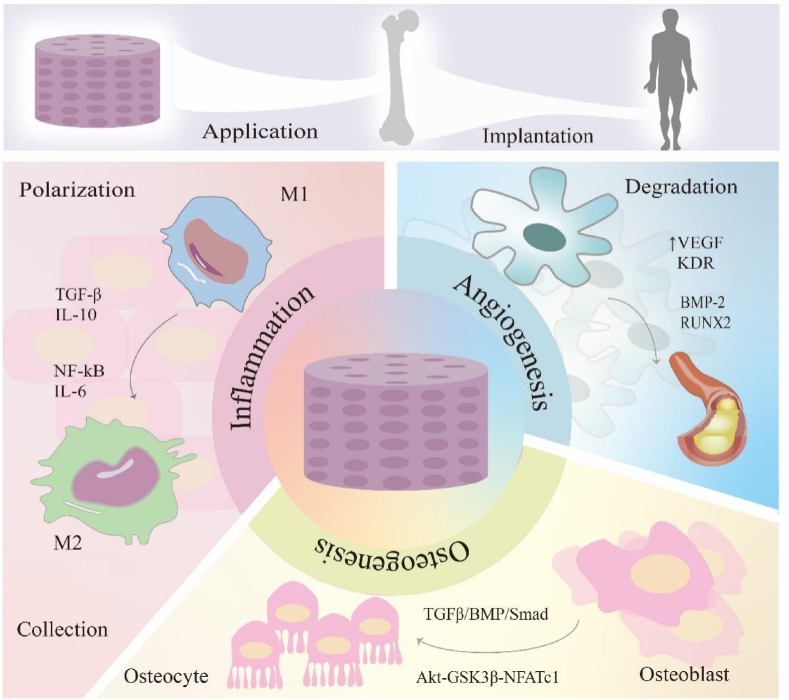
Applications of porous metal material in bone cause different kinds of reactions. Inflammation: the usage of porous metal material activates the macrophage from M1 to M2, through NF-kB and IL-6 factors. It also promotes the collection of mesenchymal stem cells. Angiogenesis: through the process of angiogenesis, porous metal material improves the basement membrane degradation by activating the amount of VEGF, KDR, BMP-2 and RUNX2. Osteogenesis: the osteoblasts are activated into osteocyte through binding of signal ways.

On that account, it is eager to find the exact evidences on the potential regulatory mechanisms. In this review, the mechanisms from three aspects are conscientiously expounded, including inflammatory response, angiogenesis and osteogenesis, as shown in Fig. 1. Aiming at providing more support and direction for future exploration, the regulations of cells, cytokines and signaling pathways by porous metal materials are particularly explored. In addition to its exceptional physicochemical and biofunctional properties, our focus extends to addressing the inherent challenges and exploring enhancement strategies, including new design methods and comprehensive improvement methods of various aspects of performance. We innovatively review the diverse signaling pathways and interactions among different cells, thereby offering researchers novel perspectives for the development of advanced biomaterials. More importantly, this review provides more technical support and optimization thoughts for applications of porous metal materials in orthopedic field.

## 2 Properties of porous metal materials

### 2.1 Structure design of porous metal material

In order to better mimic the bone marrow structure and create an immune microenvironment suitable for cell survivals, the design of porous metal materials is apparently important, including pore sizes，bioactive coatings, material categories, manufacturing methods, topographical design, and so on.

**Figure 2:**
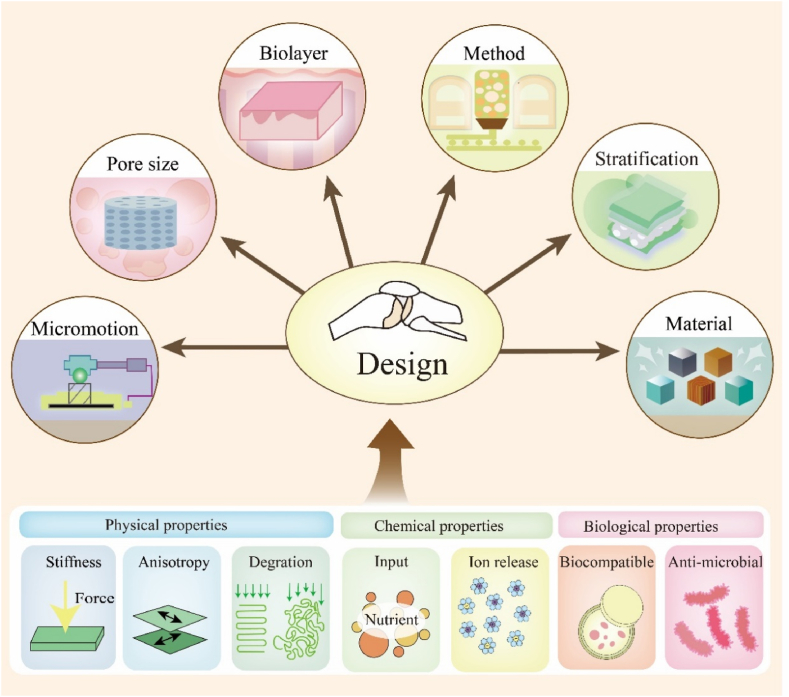
The design of porous metal materials. The design of porous metal materials is apparently important, including the pore size, biolayers, material category, manufacturing methods, stratification interface micromotion, topographical design, and so on. The aim of proper design is to make porous materials with excellent physical, chemical and biological properties, including stiffness, anisotropy, degradation, nutrition input, ion release, biocompatibility and anti-bacteria.

**Figure 3:**
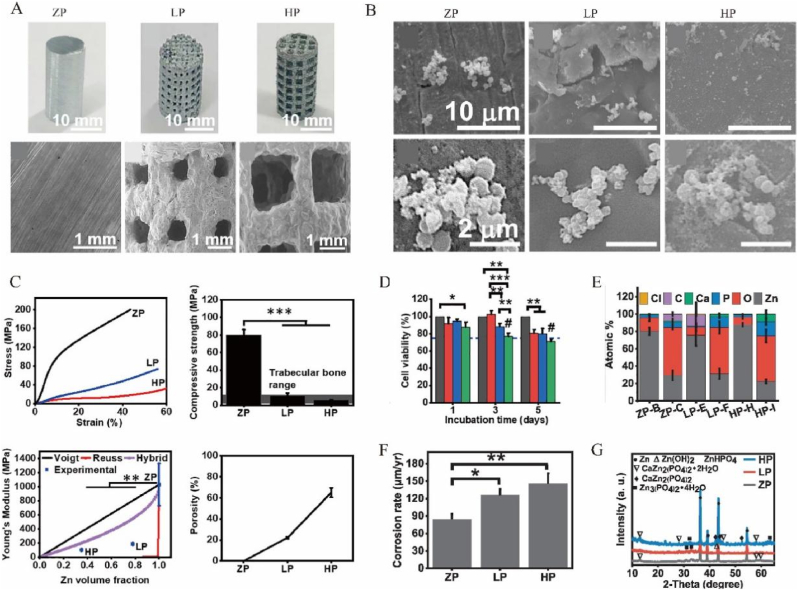
Different features of porous metal materials. **A** Digital photographs of metal scaffolds of low porosity (LP), high porosity (HP) and zero porosity (ZP) and their respective SEM images. **B** Antibacterial properties of scaffolds of ZP, LP, and HP cultured with *S. aureus* for 24 h. **C** Mechanical performance of scaffolds, including stress–strain curves at 2 % strain rate, compressive strength, rule-of-Mixtures model and the fit data, and the porosity of the respective scaffolds. **D** XRD of corroded samples. **E** MTT assay of pre-osteoblast cell viability cultured in 10 % dilution extracts prepared by incubation of samples with cell media for up to 5 days. **F** Corrosion rate taken from mass loss after immersion in Hank's solution for 1 month. **G** EDS of respective images. Reprinted with permission from Ref. [25]. © 2020 Elsevier B.V.

The pore properties of the porous metal materials can be varied, with varieties of fabricating ways (Figure 2, Figure 3A). There is a linear negative correlation between porosity and mechanical strength, i.e., an increase in porosity implies a decrease in mechanical strength. Therefore, porosity of the implant should be sized with reference to the specific implant site so that the implantation stiffness should be matched to the bone stiffness without affecting the normal physiological function. Proper porosity could lead to a suitable modulus, causing a good bearing effect and stimulating inward growth of cells [17]. Concerned with different locations of bone defects, the pore sizes change correspondingly. When it comes to skull, a high porosity of 83.5 % and a pore size of 578–642 mm meet the need for mechanical strength of the scaffold for rabbit in the upper range of cancellous bone, because it has less mechanical stimulation and requires lower stability but appropriate stiffness [18,19]. In other studies, there were also other statistics to testify it. With a pore size of 376.0 ± 11.7 μm and a porosity of 73.1 ± 6.2 %, Zn-Cu metal implant had low elastic modulus and exhibited good compressive ductility with a strain larger than 50 % [20]. It also had good plastic deformation ability, good mechanical strength and ductility, and maintained certain mechanical stability (Table 1). In the study of Ressler A. et al. [21], highly porous composite scaffolds based on chitosan prepared by freeze-gelation technique and multi-substituted CaP composite were fabricated. Large pore sizes (100–400 μm) on scaffolds are critical for promoting cell migration, angiogenesis, nutrient supply, and diffusion of metabolic waste, while small pore sizes (<20 μm) are critical for cell proliferation settlement and cell–matrix interactions (Table 1). Moreover, with gradient mechanical properties through the rational arrangement of control parameters, boundary conditions and objective function, gradient porous metal materials could decrease bone loss proportion [22]. In the study of Zhang X.Y. et al. [23], a step-wise topological design of functionally graded porous biomaterial (FGPB) ，which was manufactured from Ti-6Al-4V powder based on diamond unit cells was presented to mimic the structure of the femoral diaphysis, achieving good biomechanical performance. In the study of Zhang Y. et al. [24], the optimal pore size was 600–700 μm, which resulted in a good osteogenic response and osteogenesis ability (Table 1).

**Table 1:** Different kinds of porous metal materials designed.

Material composition	Special treatment	Pore size (μm)	Porosity (%)	Elasticity modulus (Gpa)	Compressive strength (MPa)	Refs
Zn-1Mg	Using nitrogen atomized pure Zn powder	600	67	1.17 ± 0.11	40.9 ± 0.4	[26]
Sr10-HA-PBLG	Coprecipitation method, and aminated surface modification.	30∼50	90	—	0.038 ± 0.002	[27]
Chitosan with Sr2+, Mg2+, Zn2+ and SeO3− ions	Freeze-gelation technique	Macro:100–400 Micro:<20	75	—	—	[21]
Zn-Cu	Diffusion heat treatment	296.8 ± 25.3 376.0 ± 11.7	77.6 ± 4.7	0.41 ± 0.10	12.1	[20]
Ti6Al4V	Additively manufactured using an SLM machine	Four layers		10.44	170.6	[23]
		Layer1 302	91.3			
		Layer2 574	76.9			
		Layer3 800	48.4			
		Layer4 1140	21.0			
	The three-dimensional (3D) models designed using the Pro/Engineering software	600–700	70	12.9 ± 1.1	184.8 ± 0.9	[24]
	MAO-treated	640	73	—	—	[28]
Ti	Selective laser melting (SLM) printed	600–900	60–90	1–6	40–180	[29]
Ti-Ta-Nb-Zr	Compacted and heat-treated at 250–450 °C for 5 h. Sintered at 1200 °C for 2 h in a vacuum diffusion melting furnace	166 ± 21	40	0.8	669	[30]
NiTi	L-PBF technique	500–900	80–90	10–30	63.4–77.8	[31]

Additionally, various types of bioactive coatings were incorporated to augment the cell viability and facilitated the superior osteogenic outcomes (Fig. 2). Bosco, R. et al. attached the amino-bisphosphonate drug alendronate to nHA particles, which was deposited as a thin nanoscale coating on titanium using electrospray deposition (ESD) [32]. The HA coating can improve the biological performance of bone implants in terms of bone contact and new bone formation. Moreover, the addition of layer can also improve the chemical properties. Porous titanium with porosity of ∼70 % and average macroporous diameter of ∼360 μ m was treated by AH and AO for fabricating the HA coating via an ED technique, thus obtaining a HA/L-TiO2/D-TiO2 composite coating on its surface. The scaffold could not only increase the corrosion resistance of porous Ti but also improve the osteogenic activity significantly, thus exhibiting good prospect in practical application [33].

The categories of porous metal materials also vary a lot (Fig. 2). The common metal materials used in clinic include stainless steel, titanium alloy, cobalt-based alloy, NiTi alloy and so on. B Q Li et al. prepared a porous Ti-35Nb-5Ta-7Zr alloy (wt.% hereafter) by powder metallurgical method using 50 vol% PMMA (poly methyl methacrylate) with size of 100–154 μm as the space holders [30](Table 1). Kong D. et al. utilized spherical NiTi alloy powders that were nearly equiatomic, in composition, with sizes ranging from 15 to 53 μm (d50 of 33.8 μm), to fabricate porous scaffold with pore size of 800 μm through L-PBF technique [31] (Table 1). In addition, the material ratio also plays an important role in the properties of porous metal materials [34,27] (Table 1).

The surface design on porous metallic biomaterials is also instrumental in facilitating the adhesion and differentiation of osteoblasts, thereby enhancing the integration of the implant with the host tissue [35] (Fig. 2). In the study of Civantos A. et al. [36], porous Ti was modified by directed irradiation synthesis (DIS) with argon (Ar) ion irradiation, processing conformal nanopatterning along surfaces between pores, inside pits, and along the internal walls of pores. By meticulously modulating the surface nanopatterning and nanotopography of these materials through different parameters, including energy [37], fluence [38] and incidence angle [39], DIS significantly improved the cellular interactions with the implant, optimizing the biocompatibility and functionality of the implant.

Moreover, the manufacturing method could also influence the properties of the porous metal materials. As studied, topology optimization (TO) is a powerful digital tool that can obtain optimal internal architectures for porous implants which not only meet multifunctional requirements but also mimic human bones. Ever since the 1980s, TO has been regarded as a powerful tool for implant design. Recently, additive manufacturing (AM) with layer-by-layer manufacturing, based on topology optimization, is the most promising and disruptive technology in the fabrication of porous meta implants. AM overcomes the difficulties in the advancements of porous metal materials and makes it possible to fabricate the TO-designed structure [40]. In addition, AM realized the compressive strength and elastic modulus of Zn-1Mg porous scaffolds to reach the highest [26](Table 1). AM provides more possibilities for porous metal manufacturing for medical applications [41,42]. However, most porous metal materials designed with additive manufacturing only have monotonous pore structure, such as porosity and pore size, which makes the whole structure have the same biomechanical properties, and cannot better fit the mechanical properties of natural bones as the composition of tissue exhibits significant heterogeneity. So it is necessary to study the functionally graded porous biomaterials (FGPBs) for application in orthopedics (Table 1) [23]. Except technologies based on AM, micro-arc oxidation (MAO) technology has also gradually gained popularity as an implant surface modification method because it is a convenient and efficient way to dope materials with active ingredients, change the morphology of the implant surface, and greatly enhance the biological activity of metal implants [14]. The mechanism of other elements added in the metal material can also greatly affect the functionality of the material. Yaping Wang et al. manipulated Ti6Al4V with zinc and phosphorus through micro-arc oxidation (MAO). They found that the content and proportion of phytic acid greatly influence the development of MAO coatings and the contents of Zn and P in MAO coatings [43] (Table 1).

In a nutshell, the features of porous metal materials are closely related to pore sizes, bioactive coating, material categories, manufacturing methods and topographical design. Therefore, researchers should consider the aforementioned factors in the design of porous metal materials under different requirements.

### 2.2 Physicochemical properties of porous metal materials

The research on physicochemical properties of scaffolds is an essential step in bone tissue engineering due to its key parameters that may influence the biological response and the bone healing process, such as porosity, degradability, elastic modulus, and intensity of load (Fig. 2). In the study of Cockerill I. et al., the stress–strain curves, analysis of mechanical features such as yield strength, Young's modulus, the ROM models and porosity were tested to evaluate the mechanical properties of porous zinc scaffolds [25] (Fig. 3C).

The stiffness of scaffolds is a main characteristic of physicochemical property, which should be close to that of the replaced tissue (Fig. 2). Stiffness is determined by both intrinsic elastic modulus and scaffold architecture, which can govern the macroscopic shape variables differing from the elastic modulus. At the cellular level, the elastic modulus of the scaffolds can affect the cell fate by its resistance to local deformation [44].

In clinical practice，the treatment of bone defects needs designing personalized implants to resolve various questions regarding long-term performance of patient-specific implants. A soft scaffold may fail due to the heavy deform under physiological conditioned loads. While a stiffer scaffold may cause bone resorption and implant loosening due to the stress shielding. Diverse stiffness according to the anatomical location of the tissue to be replaced is therefore a key consideration for the success of the implant [45]. Craniofacial implants emphasize on positive medical cosmetology and functional replacement, and the elastic modulus of human trabecular bone within the mandibular condyle ranges between 120 and 450 MPa [46] or within the mandible from midline to ramus ranges from 112 to 910 MPa [47]. Therefore, for the fabrication of craniofacial implants, firstly, scaffold must provide structural support for resident cells and mechanical force transduction for the craniofacial skeleton. More importantly, both plasticity and extensibility of the implant need to be considered [48]. However, for the bone under heavy stress, such as the tibia, the implant needs to have sufficient load strength and to adjust the elastic modulus according to the different anatomical position to imitate the natural bone [49].

Stress shielding is another factor that remains an unmet clinical need. Under stress shielding, a larger portion of the natural load is distributed to cortical bone, resulting in a large loss of mechanical stimuli to induce bone formation, and leading to a bone resorption over time. This will significantly impair the support force of the implant and increase micro motion of the interface between the implant and bone, giving rise to aseptic loosening and even leading to the revision ultimately [50]. In order to eliminate the stress shielding effect, implants with similar stiffness to the bone to be replaced need to be developed, which makes the anisotropy of unit cell architectures an important consideration in evaluating the overall strength of an implant. The generation of anisotropic mechanical behavior depends on microstructure of the porous arrangement. It has been reported that porous titanium showed an anisotropic mechanical behavior [51], which was produced during the manufacturing process. Meanwhile, elastic modulus, compressive strength and strain are related with spatial distribution of porous structures [52,53], which means that anisotropy should be taken into account in the profound studies. In the study of M.C. Lee [54], simulations for scaffolds with different porosities were conducted, designed as an optimal tibia defect scaffold with radial gradient porosity. It replicated a stress distribution similar to autogenous bone grafts, demonstrating that the designed gradient porosity scaffold can effectively avoid the stress shielding effect. In the simulation, the titanium alloy scaffold bore a maximum stress of 122.8626 MPa, demonstrating superior structural strength, thereby resulting in a more uniform stress distribution, reducing titanium alloy stiffness and alleviating the stress shielding effects. The proportional design of the porous metal materials should be emphatically encompassed in subsequent research endeavors, extending their application to other limbs for exhaustive comparative evaluations of their efficacies.

Another physical factor to be considered is magnetic susceptibility, especially for vivo implantation studies. Magnetic Resonance Imaging (MRI) are often used by researchers to evaluate the exact structure utilized for biomedical implants [55]. During MRI procedures, the presence of a high-intensity magnetic field can induce magnetization of ferromagnetic implants, leading to the generation of imaging artifacts and signal voids. These phenomena can obscure the visualization of surrounding tissues, thereby compromising diagnostic accuracy [56]. The causes of these artifacts are the pronounced disparity in magnetic susceptibility between the metallic implants and the biological tissues of the human body. Therefore, we should identify the factors influenced on magnetic susceptibility. In the study of Li Q. et al. [57], porous Ti–6Al–4V with a porosity of 21.7 % showed a 50 % decrease in magnetic susceptibility, and a slight decrease as the porosity increased from 21.7 % to 82.2 %. Moreover, porous Ti–6Al–4V with an irregular prism porous structure showed mechanical properties similar to those of human bone and a much lower magnetic susceptibility than that of compact Ti–6Al–4V. Furthermore, it has been noted that porous implants with a reduced metallic mass exhibit smaller artifacts [58]. All above indicate that adequate pore quality can reduce the mass magnetic susceptibility.

The changes of porosity, interconnectivity and pore size can influence chemical properties, including rate of the degradation and resistance to the corrosion (Figure 2, Figure 3). On the one hand,it may contribute to avoid a second surgery due to the materials absorbed and the tissue healed [59], and sustainable releasing of certain elements; On the other hand, a better resistance to the corrosion is beneficial to keep the stability when the implantation is asked to be permanent storage [60,61]. For example, a second surgery is often required for titanium (Ti) and Ti alloys to remove it from the body after bone healing, undoubtedly increasing the surgical risk and pain in patients. What's more, interfacial friction between the implant and osseous surfaces, coupled with wear phenomena induced by biomechanical stressors, and the biological friction corrosion effect, are inescapable contributors to the systemic liberation of titanium particulates within the peritissue environment [62]. Many studies consistently showed a significantly higher concentration of titanium particles at the inflammatory site around the implant compared to normal sites, leading to peri-implantitis [63]. The release of wear particles can have a detrimental impact on biosafety of implant and should be minimized whenever possible. However, there are some difficulties associated with the use of biodegradable metallic materials, such as dyssynchrony in the rates of metal degradation and bone healing. Furthermore, the degradation of bone-repair materials often leads to undesirable degradation of mechanical properties [63]. These cases support the view that the researchers have tended to focus on absorbing the porous metal materials rather than on the corrosion resistant materials up to now.

Therefore, when designing the porous metal implants, the effects of stiffness, strength especially anisotropy as well as porosity and magnetic susceptibility on the implant performance need to be considered simultaneously.

### 2.3 Biological properties of porous metal materials

The permeability, pore connectivity, pore size, and pore shape of porous metal implants directly affect the biological properties of implants, such as cell viability, migration and proliferation, the amount of ECM secretion, collagen gene expression [64], nutrition flow and waste removal，which consequently affect the tissue regeneration capacity of the scaffolds (Figure 2, Figure 3E–G). No matter what kind of the implants are, undoubtedly, it is essential to care about the biocompatibility, in case of the terrible effects including postoperative infection [65] and avascular skin flap necrosis [66]. The biocompatibility of porous metal materials has been widely discussed in varieties of studies, where people used either *in vitro* or *in vivo* experiments to verify the accuracy in different aspects. In the *in vitro* experiments, osteoblasts, BMSCs and other cells were cultured and observed to see their survival conditions [[67], [68], [69]]. Porous metal materials change the cell attachment patterns, allowing them to attach the implants more tightly [25,70] (Fig. 3D), while also promoting the inward growth of cells [71]. Experimental results indicated that the number of cells multiplied greatly, which means that porous metal materials had better biocompatibility than the conventional materials [72,73]. While in the *in vivo* experiments, people implanted porous metal materials into rats, rabbits as well as dogs to experience their effects [74]. Most of them showed great prognostic effect, meaning that porous metal materials have excellent compatibility *in vivo*.

Regardless of the nature of the implant, the risk of infection is high until the implant is gradually vascularized and protected by the host's innate immune defenses. However, the porous structure also indicates a significant improvement in long-term bacteriostatic activity (Fig. 2). This will help reduce the occurrence rate of severe complications in orthopedic surgery including Periprosthetic Joint Infection (PJI) [75]. After anodic oxidation surface treatments, the antibacterial rate of 3D-printed porous titanium alloy against *Staphylococcus aureus* increased from 8.5 % to 23 % and 33.75 % after one and two anodic oxidation surface treatments [76]. The antibacterial properties of zinc-doped Ta2O5 nanorods on porous tantalum surface are also outstanding, which exhibited a sterilization rate of 83.9 % against *Escherichia coli* and 95.81 % against *S. aureus* [77]. Similarly, Wang B. et al. [78] ordered 3D porous structure covered by a dense HHC36-RGD layer had a significant inhibitory effect on *S. aureus* and *E. coli*, and its antibacterial rate could reach more than 95 %. In the study of Cockerill I. et al., the antibacterial rates of the scaffolds were ∼100 %, demonstrating excellent antibacterial performance. Small clusters of *S. aureus* were found on the surface of scaffolds, their cell wall appeared to be disrupted and mineralized in many cases [25](Fig. 3B).

## 3 Influence of porous metal materials on inflammation

### 3.1 Porous materials regulate fibrin adsorption at initial stage

Upon the implantation of porous metal materials, the host initiates a complex biological response encompassing sequential yet interconnected phases of tissue injury, inflammatory reaction, proliferative regeneration, and tissue remodeling [79]. The presence of an implant modulates the trajectory and resolution of these physiological stages [80]. In the study of Xia. D. et al. [81], it was observed that four weeks post-implantation, a fibrous tissue layer had formed at the interface between the pure zinc (Zn) porous scaffolds and the osseous tissue. Progressing to the twelfth week, the newly formed bone grew into the pores of the scaffold, with a reduction in the thickness of the fibrous connective tissue layer. By the twenty-fourth week, the regenerating bone tissue demonstrated substantial integration with the Zn porous scaffolds, indicating successful osseointegration and the potential for long-term stability and function of the implant. This indicates that the absorption of fibrinogen and the formation of fibrin occurs during the initial stage of implantation and plays a crucial role in the overall bone formation.

Fibrinogen (Fg) is a soluble protein in plasma that is activated by thrombin and converted to fibrin. They not only play key roles in blood coagulation, but also exert effect on acute and reparative inflammatory pathways, affecting tissue damage, remodeling and repair. In particular, Fg has been shown to have an important role in initiating inflammatory responses to implanted biomaterials. Fibrinogen is closely related to immune cells, including neutrophil and monocyte/macrophage, etc [82,83]. Fibrinogen regulates the neutrophil function by activating pathways dependent on extracellular signal-regulated kinases. In addition, fibrinogen is tightly associated with macrophages. Fg interacts with beta2 integrin receptors, such as αXβ2 (CD11b/CD18, Mac-1) and αXβ2 (CD11c/CD18), on monocytes/macrophages. Interactions through these adhesion molecules have been reported to induce monocyte/macrophage activation. In the study of Vasconcelos, D.M. et al., pro-inflammatory molecules were downregulated and bone and angiogenic factors secreted by macrophages were upregulated [84]. Similarly, the research of Bessa-Gonçalves, M. et al., fibrinogen also promoted osteogenesis by regulating immunity [85]. FgMg extracts led to a reduction in the polarization of macrophages towards a pro-inflammatory phenotype, decreased IL-1β, IP-10, MIP-2, MDC and MIP-3⍺ [86], and promoted the expression of osteogenic markers by MSCs [85]. The novel fibrinogen-α9β1-SMAD1/5/8-RUNX2 signaling axis efficiently induces the osteogenic differentiation of hESCs and iPSCs [87].

Fg adsorption of biomaterials is rapid and substantial [88]. During the formation and degradation of fibrin clots, a variety of inflammatory mediators, such as fibrin degradation products (FDPs), can be released, which can further activate inflammatory cells [89]. Besides, fibrin clot formation can serve as a temporary scaffold that facilitates cellular activities and also deposition of a new extracellular matrix (ECM) [90]. Tarif C.M. et al. [91] designed decellularized platelet-rich fibrin (dPRF) loaded strontium (Sr) doped porous magnesium phosphate (MgP) bioceramics. In contrast of the control sample, fibrotic tissue was formed obviously at the defect site. Moreover, it achieved excellent outcomes in terms of biocompatibility, biodegradability, osteoblastic proliferation and bone regeneration. Similarly, Bessa-Gonçalves M. et al. found that during the initial stage of implantation, the fibrinogen content could be increased to form a fibrin mesh structure, and tubular connections were formed between the pores of the combination of fibrinogen and Mg-based material, which provided support for cell attachment and migration, and promoted collagen synthesis and angiogenesis. At the same time, it acted synergistically to reduce macrophage pro-inflammatory stimulation, and lead macrophages to promoting MSC osteogenic differentiation [85,86] (Table 2). In addition, fibrin glue is a biocompatible material that could be employed as a delivery vehicle in EBM-fabricated porous titanium for controlled release of BMP-2 and VEGF for improving osteogenesis of critical-sized bone defects [92].

**Table 2:** Recruiting MSCs by immune cells and their substances.

Porous metal materials	Cell line	Signaling pathway and related substance	Effects	Refs.
Fibrinogen scaffolds with Mg	Macrophage	CD86 (−)	Higher ALP activity accelerates MSCs osteogenic differentiation	[[84], [85], [86]]
		CD163 and CD206 (+)		
		TNF-α (−)		
		NF-kB p65 signaling (−)		
Immune modulating scaffold	Macrophage	CX3CR1 (+) IL-10 (+)	Enhances mRNA expression of osteogenic genes, Bmp2and ALP in MSCs. Enhances early matrix mineralization of MSCs.	[93]
Ca7MgSi4O16	Macrophage	CD80 (−)	Encourages the osteogenic lineage differentiation of BMSCs *in vitro*.	[94,95]
		iNOS, TNF-α (−)		
		CD206, Arg-1 (+)		
		IL-10 (+)		
		BMP2, RUNX2 (+)		
Porous magnesium scaffold	Macrophage	TNF-α and IL10 (+)	A higher than control cell density of hBM-MSCs.	[96]
Porous titanium implants coated with Ag nanoparticles	Neutrophils	—	A more pronounced presence of myofibroblasts and the more prominent collagen deposition reaction.	[97]
Porous titanium	Macrophage	IL-6 (−)	HMSCs survived on all surfaces	[98]
Ti6Al4V implants with AgNPs	—	CCL18 (+)	No influence on the osteogenic differentiation of hMSCs.	[99]
AH-Ti-PDA-DJK-5	Macrophage	NF-κB pathway (−) IL-1β, IL-6, TNF-α, MCP-1 (−) IL-4 and IL-10 (+)	—	[100]

### 3.2 Porous metal materials direct the interaction between mesenchymal stem cells and immune cells on acute inflammatory phase

Bone regeneration is a complex process, which involves multiple stages, including inflammation, repair, and remodeling [101]. Healing of bone fracture initiates with acute inflammation and may later transform to a regenerative or degenerative phase mainly due to the cross-talk between immune cells and other cells in the bone regeneration process [102]. Therefore, appropriate regulation of the acute inflammatory reaction is essential to initiate the healing after injury.

Mesenchymal stem cells (MSCs) are the progenitors of chondrocytes as well as of osteoblasts. In the case of a traumatic lesion, it is necessary that MSC migrate into the destroyed area, proliferate, attach to the material, and remain viable. Subsequently, they will differentiate into osteoblasts or in chondrocytes as regulated by the surrounding tissue, biomechanical stability, and growth factors [103]. The activation of osteoblasts will improve the osteogenic microenvironment, and as a result, the osteoclast function will be inhibited [104]. Therefore, inducing cell recruitment is significant.

Various reasons containing enough porosity (80–90 %) [105], and proper pore size or coating [106] of porous metal materials are beneficial to osteogenesis during this process. These factors exert the function via regulation of immune response like cell adhesion [107] and cytokine activity [108]. While, the emphasis of this part is mesenchymal stem cells (MSCs) and immune cells, especially macrophages and neutrophils [109]. Furthermore, the interaction function plays the major role in the acute inflammatory period.

#### 3.2.1 Effect of immune cell on MSCs induced by porous metal materials

Porous metal materials were previously reported to have potential ability to recruit immune cells. The healing process of bone tissue begins with the inflammation of immune cells, which releases inflammatory factors at the initial stage, recruiting mesenchymal stem cells and guiding their osteogenic differentiation [110].

Neutrophil is one of the imperative immune cells during the acute inflammatory phase, which can be raised or stimulated when implants invading or countering anti-inflammatory cytokines [111]. Researches have shown that removal of *S. aureus* aggregated from the implant surface was highly dependent on the neutrophil density [112]. If neutrophil happened to delay the recruitment, bacteria might build new biofilms to develop a tolerance to neutrophil-killing [113]. As a result, acute inflammatory phase was prolonged jeopardizing osteogenesis. Furthermore, porous titanium implants coated with Ag nanoparticles activated neutrophils, leading to a more pronounced presence of myofibroblasts and more prominent collagen deposition reaction [97] (Table 2).

**Figure 4:**
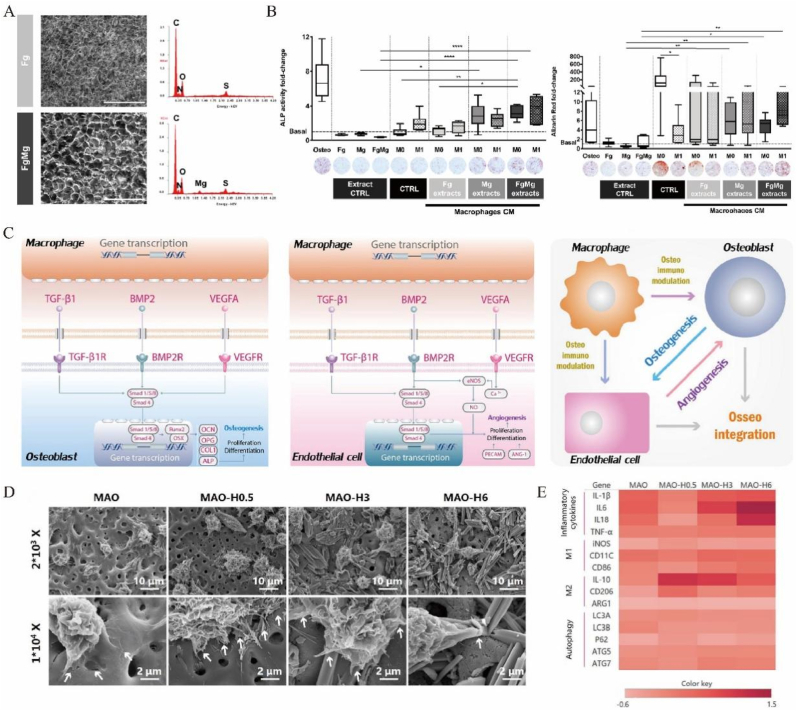
Porous metal materials regulate the inflammatory phase. **A:** The SEM image of fibrinogen and magnesium combination biomaterials modulating macrophage phenotype. Magnification:250×. Scale:400 μm. **B:** Effect of macrophage secretome on MSC osteogenic differentiation. MSC were incubated for 14 days with biomaterial extracts (extract CTRL=Fg, Mg and FgMg biomaterials extracts) or the secretome of macrophages unstimulated (M0), or LPS-IFNγ stimulated (M1), in presence/absence of biomaterial extracts (CTRL = conditioned media from macrophages not exposed to extracts; Fg, Mg and FgMg extracts = conditioned media from macrophages preconditioned with Fg, Mg and FgMg extracts alone), as indicated. Basal and osteogenic (osteo) media were used as controls. ALP and Alizarin Red staining were performed. **C:** Schematic representation of the cross-talks among MΦs to OBs and ECs. Signaling pathways of MΦs to OBs involved in the present study. Signaling pathways of MΦs to ECs involved in the present study. Cross-talks among MΦs, OBs, and ECs. **D:** Immune response of the modified surface and the interaction of bone immunoregulation, osteogenesis, and angiogenesis. FE-SEM images of MΦs morphology on samples at different magnifications. **E:** The effect of surface nano-microscopic characteristics on cytokines and interaction of bone immunoregulation, osteogenesis and angiogenesis, including the expression of OBs (BMP2, COL1, OCN, OPG, TGF-β1, VEGFA, ALP, OSX, Runx2 and SMAD1/4/5/8) osteogenic genes and genes related to angiogenesis in ECs (eNOS, VEGF and BMP2). Reprinted with permission from Refs. [85,115]. © 2020 Acta Materialia Inc. Published by Elsevier Ltd. All rights reserved. © 2018 Acta Materialia Inc. Published by Elsevier Ltd. All rights reserved.

Another important immune cell is macrophage. Porous metal materials promote the macrophage polarization and influence the cytokine release, in order to recruit mesenchymal stem cells. Wu P. et al. [114] incorporated polydopamine (PDA)-modified ceramic hydroxyapatite (PDA-hydroxyapatite, PHA) and PDA-modified barium titanate (PDA-BaTiO3, PBT) nanoparticles into a chitosan/gelatin (Cs/Gel) matrix, fabricating a porous bone scaffold. It not only effectively induced the macrophage polarization to M2 phenotype but also promoted the migration, tube formation, and angiogenic differentiation of human umbilical vein endothelial cells (HUVECs) and facilitated the migration, osteo-differentiation, and extracellular matrix (ECM) mineralization of MC3T3-E1 cells. Bessa-Gonçalves M et al. [85] fabricated FgMg compound material, which was made of fibrinogen (Fg) scaffolds combined with magnesium (Mg) discs, inhibited NF-kB p65 signaling to decrease the polarization of M1 macrophages. But it did not impact the M2 phenotypes. The extracts gradually increased the alkaline phosphatase (ALP) activity, thereby promoting the differentiation of MSCs osteogenesis (Fig. 4B). Garmendia Urdalleta A. et al. [99] detected that the 3D printed biofunctionalized Ti6Al4V implants with AgNPs suggested a decrease in their pro-repair tendency by human macrophage (Table 2). However, this response was shown to not compromise the osteogenic differentiation of hMSCs.

The above process is usually accompanied with IL-10 and IL-6. Posada, V.M. et al. [96] found that after the ion-bombardment-driven surface modification, a more notable concentration variation occurred with secretion of the anti-inflammatory cytokine IL10 after 72 h co-culture with porous magnesium scaffolds (Table 2). A higher cell density of hBM-MSCs was observed on the DPNS samples than the control after 4 h co-culture. Interestingly, related to macrophage polarization, it was also demonstrated that implantation of the 3D printed ordered Bredigite (BRT, Ca_7_MgSi_4_O_16_) (BRT-O) scaffolds increased the M2 macrophage marker CD206, meaning polarizing to the M2 phenotype. Besides, it secreted IL-10 and up-regulated the expression of osteogenesis-related genes such as BMP2 and RUNX2 [94] (Table 2). As reported, Xuan Y. et al. [116] fabricated Bredigite (BRT, Ca7MgSi4O16) bioceramic to enhance the expression of IL-10 and decrease the expression of IL-6 (Table 2). This was also proved in the study of Wang Y. et al. [100]. They introduced the DJK-5 into porous titanium to help inhibit NF-κB pathway, activate macrophage and downregulate IL-6, making HMSCs to survive on all surfaces (Table 2).

To sum up, when we talk about the immune factors working at MSCs, the topic usually is inseparable from neutrophil, the regulation of M2 macrophage polarization and interleukins such as the above mentioned IL-10. Hence, it is reasonable to promise a hypothesis that adding the related substance or material exert on immunity to deduce porous metal materials plays an anti-inﬂammatory regulatory role and promotes the progress of MSCs gathering and differentiation.

#### 3.2.2 MSCs regulate the down-regulation of inflammatory response especially through exosomes and extracellular vesicles

Generally, shortening the acute inflammatory period facilitates the osteogenesis. MSCs regulate considerable kinds of immune cells mainly by intercellular interaction and extracellular secretion including autocrine, paracrine, telecrine, and so on. Exosomes are one kind of extracellular vesicles (EVs) produced in endosomes of eukaryotic cells and have attracted attention in life sciences research and biotechnology, because of their participation in intercellular communication of various normal and pathological functions. Furthermore, exosomes are characterized by low immunogenicity, and excellent biocompatibility and biodegradability. Exosomes contain proteins, lipids, microRNA and other biomolecules. These released biomolecules can influence the immune environment, and finally change the phases of osteogenesis [117].

As shown in the previous studies, MSCs exosomes could react as the coating attaching on materials or interweave with porous metal materials [118]. First and foremost, the mechanism of how exosomes function is should be discussed. Cytokines IL-1β and TNF-α were reduced by MSC exosomes by AKT, ERK and AMPK signal pathway. Zhang et al. [119] reported that BMSCs-exosomes might promote osteogenic differentiation through the activation of the BMP-2/Smad1/RUNX2 signaling pathway, which may be one of the underlying mechanisms in bone fracture healing. Yang F. et al. [118] found that the combination of tantalum metal (porous Ta) with exosomes derived from bone marrow mesenchymal stem cells (BMSCs) could increase calcium nodule deposition, alkaline phosphatase activity and accelerate cell proliferation. Thus, BMSCs-exosomes serve an important role in improving the proliferation and differentiation of BMSCs.

Particularly, the function of exosomes might be associated with high expression of miRNAs [120], including the activation of the BMP-2/Smad1/RUNX2 signaling pathway, which may be one of the underlying mechanisms in bone fracture healing [119]. Nerve growth factor (NGF)-elicited multicomponent exosomal miRNAs activated the MAPK and PI3K-Akt signaling pathways, inducing innervated bone regeneration [121]. Additionally, porous metal materials can transport microRNAs by interweaving, leading the exosome to release related cytokines in specialized place.

On the other hand, exosomes had effect on the immune environment, which could be directly affected by the structure of porous metal materials [122]. Zhao, J. et al. [123] found that a porous Mg-Nd-Zn-Zr alloy significantly up-regulated the pro-inflammatory genes, TNF-α, iNOS and IL1β groups, compared with the control. In addition, mRNA expression of anti-inflammatory markers encoding ARG1, IL1RA and IL10 were also markedly increased. 3D-printed porous Mg-containing akermanite (Ca_2_MgSi_2_O_7_) bioceramics scaffolds effectively increased bone regeneration in cranial defects of aged rats, through exosomal-miR-196a-5p/Hoxa7/MAPK signaling axis [124]. These examples reinforce the aforementioned opinion that immune factors have influence on MSCs. Additionally, due to the properties of exosomes, it is promising to introduce the stem cell therapy.

### 3.3 Porous metal materials affect osteogenesis by influencing macrophage behavior

In the early stages of the inflammatory response, macrophages first polarize to the pro-inflammatory M1 to fight pathogens. Then, macrophages polarize to the anti-inflammatory type to assist the host in repairing the injured tissues. The characteristics of macrophage can initially determine the stage of the inflammatory response.

In the osteogenic microenvironment, macrophages polarize into different types induced by inflammatory and anti-inflammatory signals. For example, Garmendia Urdalleta, A. et al. [99] detected that the 3D printed biofunctionalized Ti6Al4V alloy implants with AgNPs up-regulated tissue-repair chemokine CCL18. Razzi, F. et al. [98] found that the 3D printed porous titanium implants with PEO treatment significantly induced lower protein levels of the pro-inflammatory cytokine IL-6 (p < 0.0001), which means that the macrophages were polarized to M2 phenotype. Other study reported that IL-4, IL-10, TGF-β, immune complexes, and glucocorticoids stimulated macrophage polarization toward the M2 type via various pathways. Porous titanium with bioactive HA on surface not only inhibited inflammation and polarized macrophages to an anti-inflammatory M2 phenotype, but also stimulated bone or angiogenesis by activating the OPG/RANKL, TGF-β and VEGF signaling pathways [115] (Fig. 4C–E). Exosomes from ASCs polarize macrophages toward an M2-like phenotype, which further enhances the exosomal proangiogenic effects. Exosomal delivery of miR-21 and positive feedback of secreted CSF-1 may be involved in macrophage polarization [125]. Therefore, macrophage polarization to the M1/M2 phenotype is a major behavior of macrophages and is a key factor to predicting the performance of biomaterials after implantation.

In addition, controlling the immune response of macrophages by changing the morphology of the implant surface can improve the biocompatibility of the implant [126].

### 3.4 Immunomodulatory signaling pathway and inflammatory cytokines during bone healing on porous metal materials

Porous metal materials can suppress inflammation by inhibiting inflammatory signaling, especially NF-κB pathway and inflammatory factor secretion, by the following concrete mechanisms.

First, the concept of immune microenvironment is introduced. The cell behavior is closely related to the immune microenvironment, for instance, the phenotypes and function of the macrophages change along with the chemical and physical queues of the environment. Chemical composition including oxygen content and pH are important factors in the immune microenvironment. Posada et al. found that the corrosion rate was notably reduced, as indicated by a slower increase in pH level post-48 h culture of the ion-bombarded porous magnesium scaffold [96]. An *in situ* oxygen release study on the porous titanium coating could exhibit the stable oxygen release and the ability to promote proliferation and differentiation of MC3T3 cell line under hypoxic conditions [127]. Li J et al. demonstrated the function of reactive oxygen species (ROS). Porous silicon nanocarriers *via* mitochondria-targeted bovine serum albumins (BSAs) generated mitochondrial ROS in macrophages for the proinflammatory transition through activation of the signaling transduction pathways including IRF5, NF-κB and AP-1 inside macrophages, via the interference with mitochondrial respiratory chains [128]. The Baicalein (BCL) treatment inhibited the activation of ROS/NF-κB signaling pathway and suppressed the production of proinflammatory cytokines such as TNF-α and IL-1 [129]. In the study of Li X. et al., ROS overproduction-induced inhibition of PI3K/AKT signaling pathway contributed to the osteoblast dysfunction and apoptotic injury on the porous titanium alloy implant under diabetic condition. Chitosan coated porous titanium alloy implant (CTI)not only alleviated osteoblast dysfunction and apoptotic injury, but also ameliorated the impaired implant osseointegration in diabetic sheep [95] (Table 2).

NF-κB signaling pathway is the most common inflammation-directed pathway, orchestrating the inflammatory response. The detailed mechanism is that extracellular ligands interact with the receptor, then activating phosphorylation IKKα/β or IKKα/α. IKKα/β may phosphorylate and degrade IκBα, releasing p65 and p50 which enter the nucleus to transcriptionally regulate the expression of target genes [130], and finally moderate the inflammation. Lower level of inflammation is beneficial to osteogenesis. Ye YJ et al. proved that the activated NF-κB signaling increased the expression of inflammatory factors such as IL-1β, TNFα, COX-2, PGE2, iNOS, NO, MMP and ADAMTS, which enhances degradations of ECM and type II collagen, and chondrocyte apoptosis [130]. Therefore, Zhao JH et al. fabricated porous Mg-Nd-Zn-Zr alloy (denoted JDBM) scaffolds coated with polydopamine (PDA). JDBM scaffolds converted macrophages from an M1-polarized phenotype to an M2-polarized phenotype, down-regulating NF-κB signaling to promote the chondrogenesis [123].

The most common way for porous metal materials to moderate the immune environment is releasing ions or other substances to stimulate the correlated cytokines. The released substances themselves may exert influence on the osteogenesis. At the same time, the altered immune factors would modulate the local microenvironment to improve the cell–implant interaction, promoting osteogenesis as well as vascularization. Materials containing strontium and strontium-based porous metal materials promoted bone regeneration and inhibited bone resorption [131]. They could also inhibit inflammation by releasing strontium ions [132,133] and improve the anti-inflammation, osteogenic and angiogenesis properties [134]. Analogously, magnesium-based materials are also widely used in the field of osteology. The released magnesium ions could promote cartilage formation and enhance cell migration [135]. More importantly, magnesium plays a role in immune regulation. In the study of Li B et al., magnesium significantly down-regulated the pro-inflammatory TNF-α and IL-1β, up-regulated the anti-inflammatory IL-10. Then, it inhibited the generation of apoptotic cells and regulated the surrounding microenvironment, which was advantageous in the recruitment of osteoblasts and promotion of vascularization [136].

In a nutshell, porous metal materials inhibited inflammation through numerous signaling pathways, and they could also attenuate the expression of pro-inflammatory cytokines.

## 4 The designed structure of porous metal implant provides positive condition for angiogenesis

The role of porous metal materials in promoting the revascularization and the angio-osteogenesis coupling of the defect site in the process of bone repair has been studied gradually. Endothelial cells (ECs) are widely involved in the whole process of angiogenesis, including basement membrane degradation, endothelial cell migration, sprouting and proliferation, and the formation of new blood vessels. However, there was a scarcity of research on the regulatory effect of porous metal orthopedic implants on the behavior of ECs. The mechanisms and pathways underlying angiogenesis are also poorly understood. This section provides a comprehensive review of the underlying mechanisms involved at various stages of angiogenesis and the regulatory effects of porous metal materials on ECs behavior.

### 4.1 The function of MMPs and degradation of basement membrane in angiogenesis

**Figure 5:**
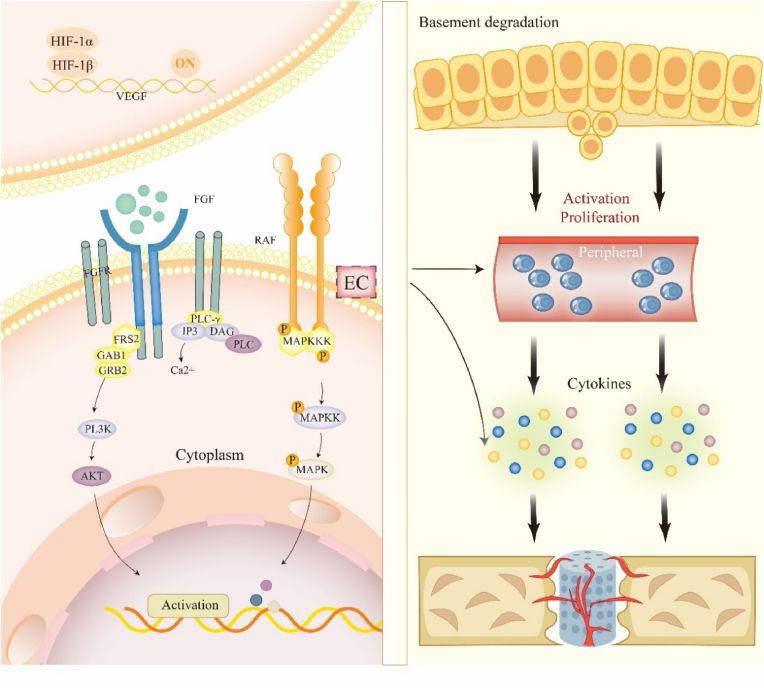
Implantation of porous metal materials activates a variety of signal pathways and cytokines related to angiogenesis. **A** Hypoxia occurs under the use of porous metal materials usually causing upregulation of HIF-a, thereby enhancing the expression of related genes. **B** Porous metal materials induce the angiogenesis of HUVECs via FGF signaling pathway. It acts on FGFR by promoting the expression of FGF to promote the activation of intracellular related cellular pathways, including RAS-MAPK and PI3K-AKT pathways [143]. **C** The first step of angiogenesis is the degradation of basement, while the release of related cytokines contributes to the proliferation of endothelial. Finally, these factors contribute to the angiogenesis.

Vascular basement membranes are primarily composed of laminins and type IV collagen to maintain them rigid and nonelastic [137]. The initial degradation of the basement membrane is a crucial step to break the physical barrier in the process of angiogenesis. During the process of angiogenesis (the development of new blood vessels), the extracellular matrix (ECM) is degraded by matrix metalloproteinases (MMPs), facilitating endothelial cell invasion and leading to sprouting of new vessels. MMPs play a role in angiogenesis during the release of vascular endothelial growth factor (VEGF) and fibroblast growth factor (FGF-2), which can maintain the viability and proliferation of endothelial cells [138]. Previous experimental studies have demonstrated the efficacy of porous metal materials in ameliorating cellular hypoxia, thus enhancing angiogenesis [139,140]. When a tissue hypoxia occurs, the secretion of angiogenic growth factors, such as vascular endothelial growth factor (VEGF), is increased, and then the endothelial cells nearby secret proteases in respond to the angiogenic growth factors to degrade the base membrane and allow endothelial cells to migrate [141,142] (Fig. 5).

### 4.2 Influence factors to stimulate angiogenesis

Angiogenesis-related factors, such as hypoxia-inducible factor (HIF), VEGF and angiogenic-related proteins (vascular endothelial growth factor and angiopoietin-1), are upregulated under the condition of hypoxia and metal degradation to stimulate the angiogenic process after implantation of porous metal materials [144] (Fig. 5).

Porous metal materials upregulate HIF-1α to activate angiogenesis. Hypoxia-inducible factor-1α (HIF-1α) gene plays a crucial role in regulating vascular reactivity, angiogenesis and osteogenesis by oxygen concentration [145]. During osteogenesis, there is a great demand on oxygen because of high metabolic of osteoblasts, forming relative hypoxia conditions. Therefore, both osteoblasts and nearby ECs may upregulate HIF-1α expression, which exerts a significant impact on energy metabolism plays a crucial role in preventing bone diseases [146]. The increased expression of HIF-1α upregulates endothelial nitric oxide synthase (eNOS), which also plays an important role in regulating endothelial function and produces endothelial cell-derived nitric oxide (NO). The NO causes vasodilation and improves blood supply to hypoxic areas. HIF-1α is also closely related to the vascular endothelial growth factor (VEGF). VEGF can promote vascular permeability, extracellular matrix degeneration, vascular endothelial cells migration and proliferation, and angiogenesis. The secretion of VEGF and the viability of HUVECs were shown to be enhanced in the presence of different materials [147,148].

The usage of porous metal materials lifts the expression of HIF-1α, VEGF and eNOS [149], which has better effects of angiogenesis and osteogenesis. For example, in the study of Zhao, H. et al. [150], porous Ti6Al4V scaffolds with a porosity of 69.2 ± 0.9 % and a pore size of 593.4 ± 16.9 μm up-regulated angiogenesis-related genes hypoxia-inducible factor 1 alpha (HIF-1α), CD31 expression and vascular endothelial growth factor (VEGF). With pore sizes of 150–400 μm and porosity of 70 %，porous calcium phosphate (CaP) scaffolds coated with aqueous soluble graphene oxide-copper nanocomposites (GO-Cu) upregulated the expression of HIF-1α in BMSCs, resulting in the secretion of VEGF and BMP-2 proteins via the Erk1/2 signaling pathway [151], which is essential for wound regeneration and tumor vascularization [152]. Similar conclusions have been drawn in the Cu-doped bioactive glass [153]. Therefore, we can speculate that the release of Cu ions can upregulated the expression of HIF-1α. What's more, hypoxia-inducible HIF-1α acted importantly in the physiology and pathogenesis of bone resorption by promoting osteoclastogenesis during fracture and influencing osteogenesis through cardiotrophin-1 (CT-1) during bone healing [154]. Ding et al. [155]fabricated a Si-doped porous TiO_2_ coating on porous metal material, stimulating the secretion of VEGF protein and expression levels of angiogenic-associated genes (VEGF, HGF, HIF-1α) in human umbilical vein endothelial cells. Peng Gao et al. [156] reported that porous Ti6Al4V scaffold performed by magnesium coating, with 68 ± 5 % in porosity and pore size of 710 ± 42 μm, increased expression of HIF-1α, resulting in enhanced VEGF at the same time. A plausible mechanism entailed that the extraction of magnesium from the magnesium-coated Ti6Al4V scaffold potentially induced an up-regulation in the expression of magnesium transporter subtype 1 (MagT1). This up-regulation subsequently led to an influx of magnesium ions [157], which in turn triggered the stimulation of VEGF transcription in human umbilical vein endothelial cells (HUVECs) *via* the activation of hypoxia-inducible factor 1 alpha (HIF-1α). This was potentially confirmed by the study of Gu Y. et al. that the Mg-TCP scaffolds designed with a pore size of about 400 μm and a filament diameter of 450 μm upregulated the expression of angiogenesis-related genes, including VEGF and eNOS [158].

### 4.3 The related signal ways on angiogenesis

What are the potential signal pathways through which porous metal materials regulate the function and state of angiogenesis? First, as mentioned above, porous metal materials upregulated HIF-1α, and HIF enhanced type H ECs, mediating local growth of the vasculature through the notch signaling [159].

More importantly, Ding Li el [143] indicated that a 6.25 % Mg-Zn-Mn alloy extract induced the angiogenesis of HUVECs *via* FGF signaling pathway. FGF signals can be transduced to the signaling cascades of RAS-MAPK or PI3K-AKT *via* FRS2 and GRB2, to the pathways of PKC or PKD through PLCγ and DAG, and to the Ca ions releasing cascade *via* PLCγ and IP3, ultimately impacting cellular proliferation and survival, and angiogenesis. They postulated that the ionic components present in the extract might exert influence on FGFR or enhance the expression of FGF to promote the activation of intracellular related cellular pathways, especially PI3K/AKT pathway. However, *in vivo* experiments are needed to furtherly confirm those *in vitro* findings. Second, in the study of Koo Y. et al. [148] the improved angiogenic differentiation was found through signal-triggered transcriptional regulation *via* the PI3K and Wnt/β-catenin pathways in the crosstalk between human adipose-derived stem cells (hASCs) and endothelial cells (ECs). Wang C. et al. demonstrated that the bioactive ions from β-CS/PDLGA composite markedly augmented phosphorylation of Akt and eNOS, stimulated NO production, and enhanced proliferation of HUVECs [160].

Except PI3K/Akt pathways, Wnt/β-catenin signaling pathway was also activated through use of porous metal materials (Fig. 5). Li B. et al. found that the 0.25 % nano-hydroxyapatite-copper-lithium (Cu-Li-nHA) composite porous scaffold promoted the angiogenic function by regulating the Wnt/β-catenin and HIF-1α/VEGF pathways which benefited to repair the Glucocorticoids-induced osteonecrosis of the femoral head (GIONFH) in the rabbit model [161].

### 4.4 Effect of porous metal materials on activity of endothelial cells

Endothelial cells have an important role in bone healing and angiogenesis. In the natural bone healing, the vascular network is established before and during the influx of osteoblasts. The establishment of vascular network is dependent on a certain number of endothelial cells. However, the presence of epithelial cells by itself is not sufficient to initiate the development of a mature vascular network and requires the migration of endothelial cells [162,163] (Fig. 5).

Porous metal materials with appropriate mechanical properties, sufficient space for bone and vascular growth, and suitable material surface morphology can promote endothelial cell viability and thus modulate the rate of endothelial cell migration, thereby reducing vascular stenosis and promoting vascular healing [[164], [165], [166]]. On the porous tantalum-composited gelatin nanoparticle hydrogel, the interaction between BMSCs-derived ECs and BMSCs could form vessels on the composite scaffold, and the co-culture of two types of cells *in vitro* could affect the maturation and stability of the vascular network [167].

The behavior of endothelial cells is also related to the thickness of the implant. Simon et al. [168] found that the cell coverage area and migration distance were significantly reduced on implants with a thickness of 75 μ m, but there was no significant change on implant with a thickness of 250 μ m. What's more, porous metal implants may also indirectly regulate endothelial cell behavior by affecting the mechanical stress. It has been shown that mechanical stimulation can affect the migration of endothelial cells. Mechanical stimulation altered the expression of pleiotropic proteins aggregated proteoglycans in human intervertebral disc cells and affected their ability to stimulate endothelial cell migration [169]. Iba and Sumpio [170] also observed that a cyclic strain regulated the ECs elongation by reorganizing the actin filaments network. Mechanical stress-induced fibroblast exosomes could regulate the proliferation and migration of the vascular endothelial cell line HMEC-1, which in turn induces the angiogenesis [171]. Multilayered Ti matrix directly regulated the cellular functions of adipose-derived mesenchymal stem cells (Ad-MSCs) and angiogenic HUVEC, and mediated their communication by enhancing paracrine effects through *in vitro* cell–matrix interactions，facilitating coupled osteogenesis and angiogenesis in bone healing [172].

The metal scaffold with extremely high internal connectivity and adequate pore size are also essential for the migration, integration and proliferation of endothelial cells [173,174].

### 4.5 Porous metal materials with pre-vascularization treatment contribute to enhancing vascular reconstruction

Classical vascularization mostly focuses on stimulating the inward growth of blood vessels into the structure of the implants, but this strategy faces the problem that the slow rate of angiogenesis of newly developed vessels leads to a long time required for complete vascularization of large implants, allowing the hypoxia time of the tissue surrounding the implant to grow, and leading to tissue defects. To solve this problem, “pre-vascularization” has been invented and has emerged as a novel and promising approach in bone implant engineering, and the deposition of pores undoubtedly help the construction of pre-vascularization [175]. The pre-vascularization materials can increase the number of blood vessels, the construction of vascularized tissue [167] and the velocity of bone regeneration [176]. The ideal pre-vascularized tissue should have a hierarchical structure as the native vasculature in order to align with the host vasculature. After implantation, the vascular network could be quickly anastomosed to the host vascular network system, and the survival rate of the implant could be improved to a greatest extent [177]. One of the ways to achieve pre-vascularization is seeding of angiogenic cells onto scaffolds *in vitro*, such as human umbilical vein endothelial cells [178] and endothelial progenitor cells. For example, in the study of Xu J. et al. [179], β-tricalcium phosphate (TCP) scaffolds with pre-vascularization was performed in the muscle pocket created within the vastus lateralis muscle and then implanted into the tibial defect model. The mRNA and protein expression of bone morphogenetic protein 2 (BMP2) and vascular endothelial growth factor (VEGF) were significantly up-regulated during bone regeneration, indicating that the *in vivo* pre-vascularization strategy can effectively enhance the vascularization and osteogenic ability of β-TCP scaffolds in bone defect repair. It is worth noting the osteogenic and angiogenic genes expressions of BMSCs co-cultured and seeded on P600 porous scaffold were significantly higher. Lin et al. found that the design of arrayed hollow channeled silk scaffolds with human umbilical vein endothelial cells (HUVECs) promoted the survival of pre-seeded bone marrow stem cells (BMSCs) *in vivo* [178]. This provided an engineered vascular bed and facilitated the formation of micro-vessels and vasculatures with both human and mouse origin. According to the above descriptions, it can be proved that pre-vascularization can better improve the biocompatibility of implants, and further studies in this aspect need to be carried out in the future.

## 5 Porous metal materials play different roles in the process of osteogenesis

### 5.1 Different pore quality play different roles in cell adhesion and proliferation

Porous metal materials have a significant promotion effect on cell proliferation. Their special three-dimensional structures could significantly increase the adhesion rate of cells [180] and the deposition of extracellular matrix (ECM) [181]. With regard to pore quality, we usually talk about the pore size, connectivity and pore geometry.

In the study of Bie H. et al., hydroxyapatite (HA)- zirconia (ZrO2) - polyvinyl alcohol (PVA) composites with porosity of close to 65 %, can better facilitate the material transfer between cells on account of the existence of the holes, achieving the growth of chondrocytes and osteogenesis [182]. In the study of He J. et al., with pore sizes of 474 ± 103 μm and a porosity of 93.4 ± 0.3 %, the porous 20Fe@40Zn scaffold showed that the ratio of the new bone volume over total volume (BV/TV) of group was 25.6 (±5.9) % and the bone-to-implant contact (BIC) was 82.9 (±3.9 %) in the quantitative analysis. These newly formed bone tissues did not directly bond to the scaffold skeletons but were separated by a layer of connective tissue [183] (Fig. 7C–E,F). Kingsak M. et al. [184] used a controllable titanium dioxide (TiO_2_) with nanotube array to explore the influence of pore. Cell adhesion was significantly enhanced by controlled nano-topographies of TiO_2_ nanotube arrays with 80 nm of pore size. Moreover, referring to better cell diffusion and significantly larger cell area coverage, TNAs with smaller pore sizes (TNA30 and TNA80) took advantages. Li C. et al. [185] found that in the 3D-printed PLGA/β-TCP/Zn scaffolds, the internal pores were connected, the large pore size was about 506.6–531.3 μm and the porosity was ranging from 66.4 % to 68.2 %, which was favorable for osteogenesis. At the same time, it was observed that after adding Zn-SPs, many micropores with diameter under 1 μ m were generated [185]. This multi-level pore structures and porosity are similar to the native cancellous bone [186], and were favorable for BMSCs ingrowth and proliferation, and osteogenic differentiation [161].

**Figure 6:**
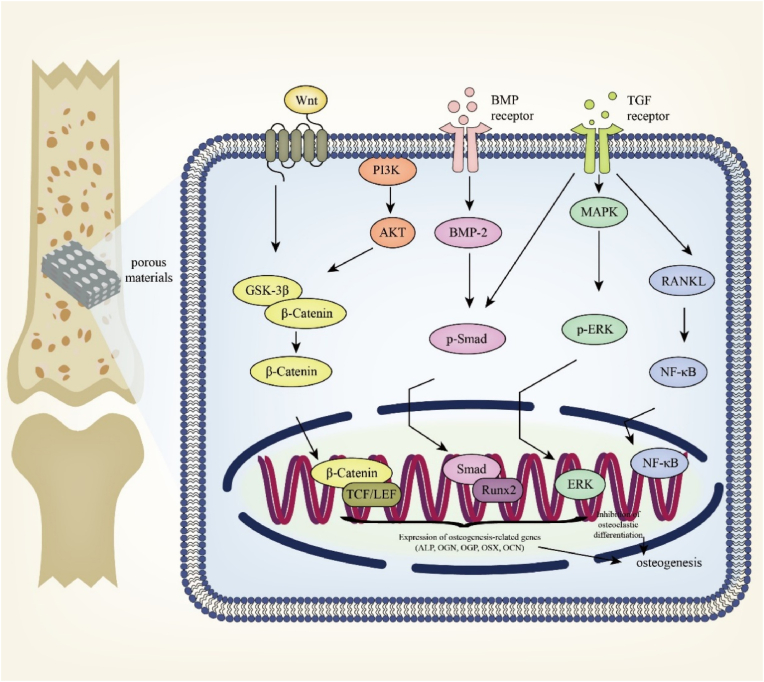
Influence of porous metal materials on osteogenesis. The degradation of porous metal materials causes the dispersed distribution of metal ions, which causes the converge of osteoblast. BMP enters the osteoblast and makes the phosphorylation of Smad, which inhibits the expression of RUNX2 and up-regulates ALPL. The phosphorylation of GSK3β triggers β-linked catenin, then promotes the expression of Wnt, which finally promote the expression of RUNX2. It also improves the AMPK signal pathway, which improves OGN, OGP, ALP, OSX, OSN and OCN, linking to osteogenesis. The activation of AMPK also inhibits mevalonate, promoting the differentiation of osteoblast.

**Figure 7:**
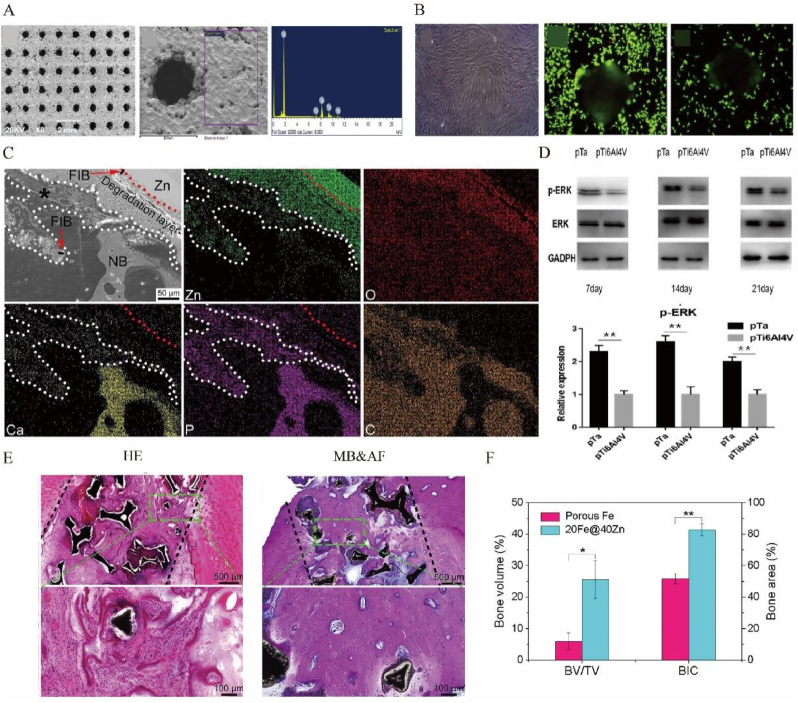
Porous metal materials act important part in osteogenesis. **A** Scanning electron microscopy was used to observe the pore size of the two materials, and the energy spectrum was used to analyze the surface composition of the material. **B** Adhesion of BMSCs. Light microscopy image of BMSCs and fluorescence microscopy image of live/dead cell staining of BMSCs cocultured with the porous tantalum and pTi6Al4V for 1 day. **C** Q-PCR showed p-ERK and ERK protein expression of BMSCs cocultured with pTa and pTi6Al4V for 7, 14 and 21 days, GAPDH as an internal reference. **D** SEM images and EDX elemental maps of the bone sections with 20Fe@40Zn scaffolds after 3 months of implantation. They showed new bone and connective tissue layer surrounding the skeletons of the scaffold. **E** Histological analysis of the bone tissue around the porous 20Fe@40Zn scaffolds. **F** Quantified BV/TV and BIC from the micro-CT reconstructions. Reprinted with permission from Refs. [183,187] © 2019 The Authors. Published by Elsevier (Singapore) Pte Ltd on behalf of Chinese Speaking Orthopedic Society. © 2020 Acta Materialia Inc. Published by Elsevier Ltd. All rights reserved.

Geometry and surface area are also significant influencing factors. In porous NiTi alloy implants, cell adhesion and growth on porous NiTi alloy samples were both negatively correlated with the M-average pore size of the samples. This is due to the fact that porous materials with smaller pore sizes have a higher S/V ratio (specific surface area), which allows more cells to attach [188].

In light of above instances, we may find that pore characteristics do significantly affect the cell adhesion. But when material types are different, their influential factors and specific values vary from each other. Interestingly, there are some common regularities. It was illustrated in the study of Luo Y. et al. [189] and Zhu Y.L. et al. [162], the cell adhesion peaked at intermediate values of porosity. To clarify, their research for porous hydroxyapatite (HA)/BaTiO_3_ piezoelectric composites with porosity between 40 and 60 % and zirconia scaffolds with porosity between 92.7 and 68.0 %, the cell adhesion was best at porosities of 50 % and 75 %.

The effect of pore quality on the external cell matrix also needs to pay attention. Extracellular matrix (ECM) has good biological activity, especially cell adhesion, which has been proved in the study of W. Cui et al. [186]. The constructed SIS/SrFeHA scaffolds characterized by highly porous structures, rough micro-surface and improved mechanical strength, efficiently released the of bioactive Sr^2+^/Fe^3+^ and ECM component. The cell growth rate was higher, the ECM covered almost the whole surface of the scaffold, and the cell adhesion rate was much higher. The ECM needs to be deposited on the matrix for cell adhesion before the cells can attach. Appropriate pore size and porosity could facilitate the deposition of ECM [190].

### 5.2 Porous metal materials regulate formation and function of osteoclasts

Currently, there is a lack of researches on the effect of porous metal scaffolds on osteoclast adhesion. Therefore, we mainly focus on how porous metal materials influence the formation of osteoclast and the crosstalk between osteoblasts and osteoclasts.

Under porous metal materials, the default differentiation of bone marrow stromal cells (BMSCs) is skewed towards adipogenesis and osteoclastogenesis, exacerbating bone loss through increased resorption and decreased formation [191,192]. The osteoclastogenic process is predominantly regulated through the interplay of receptor activator of nuclear factor-κB ligand (RANKL) and osteoprotegerin (OPG), with additional modulation by pro-osteoclastic cytokines such as interleukin-6 (IL-6), tumor necrosis factor-alpha (TNF-α), and macrophage colony-stimulating factor (M-CSF) [193]. RANKL, produced by osteoblasts, binds to its receptor RANK on the surface of osteoclast precursors, thereby triggering intracellular events that lead to the activation and maturation of osteoclasts. In experiments performed *in vitro*, the expression levels of RANKL in OP-BMSCs are often used to detect the effects of osteoclast genesis.

Various studies conducted the effects of porous metal materials on osteoclasts. In general, porous metal materials can inhibit osteoclast activity to achieve a balance between bone formation and bone resorption. In the study of Lin S.J. and C.C. Huang, a multifunctional strontium peroxide (SrO2)-based oxygen-generating scaffold was found to disrupt the organization of the actin ring, which is required for their bone resorption activity in osteoclasts, suggesting the suppression of osteoclast activity. Furthermore, the formation of osteoclasts were reduced as well [194]. The inhibition effect was reduced when CMS was absent from the scaffolds (GO/SF). In the study of Yang X.J. et al., by quantifying the size of the resorption pit on the osteo assay surface plate, the bone resorption activity of a novel zoledronic acid loaded gelatin nanoparticles integrated porous titanium was evaluated. The formation of resorption pits by RANKL-induced osteoclasts were significantly inhibited [195].

Moreover, considering the special microenvironment of bone defect, it is necessary to block its excessive osteoclastic activity. Osteoprotegerin (OPG) is a glycoprotein belonging to the tumor necrosis factor (TNF) receptor superfamily, which is considered to be a factor that inhibits bone resorption. OPG is mainly expressed by osteoblasts, which inhibit osteoclastogenesis and osteolysis via binding and neutralizing RANKL, through RANKL/RANK/OPG system [196]. It is well known that the level of Runx-2 and OPN reflects a bone-regenerating environment. In the study of Wang X. et al., the fluorescence intensity analysis of Ti6Al4V scaffolds demonstrated that the existence of OPG reduced RANKL expression levels, thus inhibiting osteoclast activity. The cooperation of BMP-2 and OPG decreased RANKL to a higher degree compared with OPG loading alone. The synergistic release of OPG enhanced the osteogenic activity, inhibited the osteoclastic activity around the osteoporotic bone interface, and finally promoted the osseointegration of the 3D-printed composite scaffolds [197].

Sr ions is closely related to the expression of osteoblast–osteoclast coupling factors. In the study of Chen F. et al., porous strontium-substituted biphasic calcium phosphate (Sr-BCP) also mediated the expression of important osteoblast–osteoclast coupling factors, as evidenced by the increased OPG/RANKL ratio in mMSCs, and the reduced Rank expression and enhanced EphrinB2 expression in osteoclast precursors. Sr-BCP could clearly accelerate the ectopic bone formation by promoting osteogenesis but suppressing osteoclastogenesis [198]. Sr^2+^ in a dose-dependent manner can inhibit differentiation of osteoclasts, and promote apoptosis of mature osteoclasts to decrease bone resorption [199,200]. In the study of Yu S. et al. [201], the expression levels of TRAP, matrix metalloproteinases-9 (MMP-9), and cathepsin K (Ctsk) was significantly reduced after treatment by Sr/Mg@HA/PLGA-CAS, proved the effect on preventing RAW 264.7 cells induced by RUNKL from differentiating toward osteoclast [201].

### 5.3 Porous metal materials promote osteogenesis through different pathways

#### 5.3.1 Porous materials promote bone formation through inhibiting the NF-κB pathway

Porous metal materials can affect osteogenesis by NF-κB pathway. NF-κB pathway plays an important role in the mid-osteogenesis and joint tissue by regulating the activity of synovial cells, osteoblasts and chondrocytes [202] (Fig. 6). It can also exert effect on inflammatory response that has been discussed in Section 3 [130,[203], [204], [205]]. Notwithstanding the fact that NF-κB pathway has multiple functions, the following part focuses on how porous metal materials can promote osteogenesis by the NF-κB pathway.

Initially, it has aforementioned that the inhibition of NF-κB pathway promoted the transformation of macrophage phenotype from M1 to M2 [206]. Inspiringly, Bessa-Goncalves M. et al. [85] suggested that this immune action could further promote osteogenesis. Their study utilized fibrinogen (Fg) and magnesium (Mg) in a combination (FgMg). Because Fg and Mg have synergistic effect on macrophages, the combination extract highly inhibited the activation of NF-kB pathway when meeting M1 stimulating. Furthermore, NF-kB inhibition associated with p65 silencing could promote the polarization of M1 to M2. According to the control test, it was found that the polarization of regenerative phenotype induced ALP activity and mineral deposition on MSC by macrophage products, which was beneficial to osteogenesis [207].

Zhao J. et al. [123] demonstrated that porous Mg-Nd-Zn-Zr alloy scaffolds coated with poly dopamine (PDA) could both convert M1-polarized macrophages into M2-polarized macrophages and have a protective effect on cartilage by downregulating the expressions of MMP1, MMP3 and MMP13. Wang Y. et al. [100] also found that porous Ti alloy with Herein and DJK-5, a class of host defense peptides (HDPs) had a positive inhibitory effect on osteoclastic differentiation under the condition of inflammatory environment *via* operating the release of pro-inflammatory and anti-inflammatory factors, as well as impeding the activation of NF-κB pathway, on the account that both NF-κB P65 and IKKβ expressions were significantly decreased in the RAW264.7 grown on the AH-Ti-PDA-DJK-5. NF-κB P65 and IKKβ as the mean factors of NF-κB pathway were also greatly reduced [100]. Blocking the transcription factor NF-κB inhibits RANKL-induced osteoclast generation and keeps macrophages active. As for the function of osteoclast, another study was similarly demonstrated. Osteoclasts could differentiate and degrade the Fg-3D, and produce TGF-β1 and other factors that promoted osteogenic differentiation of MSC [208].

In general, the use of porous metal materials can inhibit the NF-κB pathway to promote osteoclast formation. However, due to different design methods of porous metal materials, they can also activate the NF-κB pathway to promote osteoclast formation, leading to better osteogenic effects. Wang S. et al. [209] found that Zn^2+^ simultaneously promoted the expression of p-IκBα and p-p65, while the osteoclast-specific genes RANKL and RANK [186] were upregulated, indicating that NF-κB signaling was activated during the process of osteoclastogenesis. They demonstrated that activation of the NF-κB pathway was a potential mechanism underlying the osteoclastogenesis induced by Zn^2+^.

In a nutshell, change of NF-κB pathway through promoting immune cell differentiation plays a significant role in promoting bone formation.

#### 5.3.2 Other related transduction pathways in osteogenesis mediated by porous metal materials

There are a variety of transduction pathways which can regulate different cell behaviors, angiogenesis and osteoclast formation through different pathways (Fig. 6).

The Wnt signaling pathway, especially the canonical (Wnt/β-Catenin) signaling pathway, is one of the most pivotal signaling pathways in the process of angiogenesis and bone regeneration and repair [210]. Li B. et al. [161] found that porous copper- and lithium-doped nano-hydroxyapatite composite scaffold could increase the expression of osteogenic and angiogenic factors along with the activation of factors in Wnt/β-catenin *in vitro* and *in vivo*. Moreover, Li C. et al. [185] found that, with 3D-printed PLGA/β-TCP/Zn scaffolds, the expression of four proteins (BCL9L, FZD2, ENPP1 and HTRA1) directly related to β-catenin was up-regulated by proteomic analysis. Among the four proteins, the increased activity of FZD2 could explain the activation of Wnt/β-catenin signaling pathway [211]. Furthermore, ENPP1 and HTRA1 have been shown closely related to osteogenesis and were presented downstream of Runx2 [185]. All the above studies are reliable to approach the viewpoint that porous metal materials can improve osteogenesis by up-regulating Wnt/β-catenin pathway [161].

The phosphatidylinositol 3-kinase/protein kinase B (PI3K/AKT) pathway plays significant roles in regulating the cell cycle, promoting the differentiation of osteoblasts and regulating the balance of bone metabolism. Meanwhile, PI3K/AKT pathway interacts with other pathways, such as the above Wnt/β-Catenin pathway. Akt can stabilize β-catenin by activating GSK3β, thereby promoting the activation of Wnt signaling pathway [212]. Porous scaffolds are considered to be good biocompatible and osteoconductive materials that positively modulate the cell–material interactions [213]. Therefore, porous materials could regulate osteogenic differentiation through Akt/GSK3β signaling pathway and through different downstream in multiple ways [214]. Xu H. et al. [215] confirmed that Piezo1 porous metal material sensed to mechanical stress and regulated macrophage responses through the AKT/GSK3β signaling pathway and its downstream factor-Ccnd1 cell cycle protein. Meanwhile, Zhu D.Y., fabricated gadolinium-doped bioglass porous scaffolds, with pore size of mainly around pore sizes of ∼150 μm. The addition of gadolinium promoted the osteogenic differentiation of hBMSC and facilitated bone repair *in vivo* through the Akt/GSK3β pathway [213,[216], [217], [218]]. The activation of the Akt signaling pathway inactivated GSK3β by phosphorylating Ser9 of GSK3β, which resulted in the accumulation and translocation of β-catenin, thereby mediating the upregulation of ALP, OCN and BSP, finally promoting the regeneration of bone. Tan J. et al. [219] found that Ti45NB, a kind of Ti–Nb alloys could accelerate the formation of new bone, enhance the osteogenic differentiation of MC3T3-E1 cells and upregulate remodeling efficiency, resulting in a better recovery of early mechanical stability of the fractured femur compared to the Ti6Al4V control. There was a higher expression of PI3K and Akt in the Ti45Nb group in histological staining at weeks 4 and 8 and significantly at the callus, indicating a role in the endochondral ossification of the callus. Therefore, the mechanism by which Ti45Nb promotes fracture healing involve the PI3K/Akt signaling pathway. This fits with the study of Zhang H. et al. [220] on the porous micro/nano morphology-controlled bioceramic scaffolds. What's more, Zhang Q. et al. [221] confirmed that the monotropein (Mon) inhibited lipopolysaccharide (LPS)-induced bone loss *via* the Akt-GSK3β-NFATc1 signaling pathway, and also inhibited LPS-induced phosphorylation of Akt and GSK3β and the nuclear translocation of NFATC1 in OCs from BMMs, and these inhibitory effects of Mon were reversed by the AKT agonist SC79. It also inhibited LPS-induced osteoclast formation *in vitro*, which was proved by study of Lu S.H. et al. [222]. Moreover, significant enrichment in PI3K-Akt signaling pathway was revealed in the KEGG analysis, under the culture of amino-functionalized zirconium-based metal–organic frameworks [223]. The positive genes in the PI3K-Akt signaling pathway increased significantly, like Sgk1, Spp1, and integrin subunit α 5 (Itga5). Itga5 belongs to the integrin α chain family that functions in cell surface adhesion and signaling [224]. The scaffold achieved a great effect on bone tumor treatment and osteogenesis promotion. The above evidences suggest that the Akt/GSK3β pathway can play a key role in the promotion of bone regeneration by porous scaffolds.

Porous metal materials can also regulate osteogenic differentiation through the TGFβ/BMP/SMAD-relate signaling pathway and in multiple ways through different downstream. TGF-β1 could be added to the surface of porous metal materials to influence the repair [225] or BMP-2 could be added to the scaffolds to promote osteogenesis [[226], [227], [228]]. For example, BMP-2-coated BSA nanoparticles were prepared by dissolution technique, and then coated with CS and oxidized alginate to achieve slow release of BMP-2, directionally inducing the osteogenic differentiation of MSCs [227]. It was even possible to put in TGFβ and BMP-2 at the same time [229]. In addition, different porous metal material can be designed to promote osteogenic differentiation *via* the BMP-SMAD pathway. Ma X.Y. et al. [230] fabricated nanophase HA/CS composite coated porous titanium implants (nCT). The focal adhesion kinase (FAK)-mediated BMP-2/SMAD pathway played a role in mediating the promotive effect of nCTs on osteoblast adhesion and differentiation under diabetes-induced high reactive oxygen species (ROS) condition. In the study of Huang Z. et al. [231], the magnetic iron oxide/polydopamine coating improved the osteogenesis of the 3D printed porous scaffold by promoting bone formation through the promotion of the SMAD pathway. The expressions of bone morphogenetic protein (BMP-2), phosphorylated Smad1/5 and Runx2 were significantly up-regulated at the protein level. Runx2 is the downstream regulatory molecule of Smad1/5, thereby upregulating the COL1, OCN, Runx2 and ALP. A similar point was made in the study of Wu T. et al. for cps-pdms scaffolds [232].

ERK/AMPK signaling pathway is another significant way and in multiple ways through different downstream. It has been shown that the nano-structured materials can synergize with exogenous cytokines to have a significant impact on human osteoblast differentiation [233] (Fig. 6). Dou X. J. et al. [187]found that porous tantalum promoted osteogenic differentiation of bone marrow MSCs *in vitro* through the MAPK/ERK signaling pathway. By increasing the expression of its effector protein p-ERK, the expression of osterix (OSX), collagen-I (Col-I), osteonectin (OSN) and osteocalcin (OCN) and other osteogenic genes were upregulated, promoting bone formation and differentiation of BMSCs *in vitro* (Fig. 7A, B, D). Similar consequence was seen in lanthanum-doped mesoporous bioglass/chitosan composite scaffolds [234]. Moreover, the titanium particles significantly affect phosphorylation of ERK1/2, a key component of MAPK signaling. This suggests that the MAPK signaling pathway is involved in the inhibition of osteogenic differentiation of rMSCs by titanium particles [235], showing that the release of wear particles can have a detrimental impact on implant biosafety. According to the study of Jiang T. L. et al. [236], porous metal materials coated with magnesium tricalcium phosphate on surfaces upregulated the proliferation of bone-derived osteoblasts through Shc and ERK1/2 MAPK signaling.

The Hedgehog signaling pathway also plays an essential role in various developmental processes, such as osteoblast proliferation, differentiation, and maturation as well as bone formation [237,238]. The Hedgehog signaling family encompasses three critical protein ligands, Sonic Hedgehog (Shh), Indian Hedgehog (Ihh), and Desert Hedgehog (Dhh), which signal via a sophisticated mechanism involving transmembrane proteins, including Smoothened (Smo) and Patched homolog 1 (Ptch1) [239]. Upon a Hedgehog protein ligand binding to Ptch1, Smo transform into a persistently active state, thereby initiating an intracellular signal transduction cascade. This cascade culminates in the enhanced transcription of target genes such as Gli1, which plays an important role in the balance of bone resorption and formation. Several studies have indicated the potential role of the hedgehog pathway in mediating the response of osteoblasts to biomaterial topographies. In the study of Lin Y. et al., the micro-/nanotextured topography (MNT) titanium achieved better osseointegration *in vitro* and elevated effect on osteoblast proliferation and differentiation, induced higher gene and protein expression of Shh, Smo, and Gli1 and the activation of Hedgehog signaling compared to smooth and microstructure surfaces [240]. However, the addition of porous structures can achieve better effect than scaffolds with only nanostructures. In the study of Gao Y. et al., [241] the effects of micro/nano-porous zirconia on adhesion, proliferation, osteogenesis and angiogenesis of human bone marrow mesenchymal stem cells were enhanced. FGFR3 is a upstream negative regulator of hedgehog signaling [242]. The porous structure inhibits expression of FGFR3, promotes activation of hedgehog signaling and finally upregulates osteogenic gene expression in hBMSCs [241]. Moreover, Hedgehog signaling interact with various signal pathways [243], including BMP [244] and Wnt signaling pathway [245], conjointly playing a modulatory role in the osteoblast proliferation and differentiation process.

## 6 Conclusions

This article summarizes the current progress achieved in the mechanism of porous metal materials promoting bone healing, including inflammation, angiogenesis and osteogenesis. Holding the balance is a key problem for porous metal material design. Through changing the behavior and state of different cells, including macrophages, osteoblasts and osteoclasts, porous metal materials can activate different pathways and release regulatory factors, thus playing an important role in improving the bone healing effect. The inflammatory reaction balance should be grasped, and the excessive or insufficient inflammation should be avoided. At the early stage of implantation, the porous metal material regulates the acute inflammatory period, then promotes the polarization of macrophages achieving the phenotype to anti-inflammatory state, which is conducive to the process of osteogenesis and angiogenesis. Angiogenesis and osteogenesis are interdependent processes, but their interaction is very complex, so it is necessary to achieve the optimal synergistic effect in the design of scaffolds. At phase of bone repair, bone formation and angiogenesis are inseparable in parallel. The forming blood vessels bring nutrients and necessary oxygen to the repairing bone, while osteoblasts can also secrete molecules that promote angiogenesis in turn. Porous metal materials greatly promote the interaction of osteogenesis and angiogenesis by promoting the secretion of VEGF by osteoblasts and regulating the behavior of endothelial cells. It also affects the interaction between immune cells, osteoblast-related cells and endothelial cells, thus showing excellent performance in bone repair and angiogenesis. Furthermore, the conceptualization of pre-vascularized porous matrices offers a particularly promising perspective for enhancing osseous regeneration. Ultimately, the employment of metallic scaffolds with optimized porosity facilitates superior osteo-remodeling through the activation of NF-κB pathway, Wnt/β-Catenin pathway, PI3K/AKT, TGFβ/BMP/Smad-relate pathway and ERK/AMPK pathway. If the material is biodegradable, the degradation rate of porous metal stents need to match the cycle of bone repair. The excessive release of ions may lead to toxicity problems. In addition, the mechanical properties of the scaffold should match those of the host bone tissue.

The design of porous metal materials is undoubtedly diversified, with its varying pore sizes, porosity material types, modification methods and coatings help researchers create the most experimentally suitable and clinically effective scaffolds. However, according to the literature reviewed, the diverse design and serried mechanism of porous metal materials are very complex, having the function of regulating different cell types, numerous regulatory factors, and intertwined signal networks, which brings difficulties to the review. Current understandings of the mechanisms tend to emphasize on cellular regulation of immune cells, angiogenesis, osteogenesis and osteoclast functions. They provided a theoretical basis and insight for the application of porous metal materials in orthopedics. However, beyond its superior physicochemical and biological properties, we also focus on the challenges faced by porous materials and the methods for their improvement. This includes the incorporation of novel design approaches and a comprehensive integration of enhancement strategies for various performance aspects. We have reviewed the distinct signaling pathways and the interconnections between different types of cells, providing researchers with new perspectives for the development of innovative materials. We found that despite many different design methods, porous metal materials still had internal consistent laws. Furthermore, most studies have been done *in vitro* with one type of cell. To better simulate cellular effects *in vivo*, crosstalk between two or more cell types is worth investigating. The biological effects and mechanisms of magnesium alloy implantation *in vivo* need to be further explored and verified. In addition, at present, the design of porous materials mostly stays in the *in vitro* or animal experimental stage, while the ultimate purpose of studying porous metal materials is to enable them to be eventually applied to the clinic and serve patients, so we need more vivo validation tests. Thus, the porous metal material can be continuously optimized to design the most suitable material.

## Declaration of competing interest

All authors declare that there are no competing interests.
